# Trans Expertise and the Revision and Translation of ICD-11: An Analysis of Available French Data from a Trans Perspective

**DOI:** 10.3390/ijerph191911983

**Published:** 2022-09-22

**Authors:** Anna Baleige, Mathilde Guernut, Frédéric Denis

**Affiliations:** 1 EA 75-05 Éducation Éthique Santé, Faculté de Médecine, Université François-Rabelais Tours, 2 Boulevard Tonnellé, 37044 Tours, France; 2 UMR 8163 Savoirs, Textes, Langage, CNRS, Université de Lille, Rue du Barreau, BP 60149, 59653 Villeneuve d’Ascq, France; 3 Service d’Odontologie, Centre Hospitalier Universitaire de Tours, 2 Boulevard Tonnellé, 37044 Tours, France

**Keywords:** transgender persons, terminology as topic, knowledge, community-based participatory research, International Classification of Diseases

## Abstract

Transgender and gender diverse (TGD) individuals’ depsychopathologization in the eleventh revision of the International Classification of Diseases (ICD-11) faces systemic discriminations built-in epistemic pipelines. Based on an analysis of unexploited data from ICD-11 and the French translation process, this article addresses power issues in participatory research and systemic discrimination within a socio-cultural context. We used a peer-driven participatory approach to conduct qualitative analyses of the French version of the ICD based on contributions from 72 TGD participants in the French study for ICD-11. The results highlight a major incongruence between participants’ propositions and the final official translation. Alternative terms were proposed and discussed by participants in regard to usage and concepts, but also encompassed participation and perceived futility of maintaining pathologization. We found discrepancies in the French publication and translation processes, respectively on gender categorization and back translation. These results question the relevance and implementation of ICD-11 for TGD communities and highlight failures at all three stages of the official French translation. Power issues have an impact on knowledge production and, while mechanisms vary, all relate to epistemic injustice. Involving TGD communities in all stages of medical knowledge production processes would reduce transphobic biases. Individuals with personal stakes involved in politicized research areas appear all the more necessary today.

## 1. Introduction

Transgender and gender diverse (TGD) [1] communities, especially TGD youth, face growing discrimination in accessing services. Systemic discriminations have a direct impact on public health policies by shaping both political and scientific debates [2]. This follows the publication of the eleventh revision of the International Classification of Diseases (ICD-11) and its epistemic changes which made a health policy redesign necessary [3]. TGD health appears to be at a critical juncture, in terms of public policies, which will shape future health practices and behaviors. It is also an opportunity to demonstrate the impact of scientific knowledge production in health debates.

Recent events in France have highlighted the significant influence of transphobic lobbies in parliamentary debates and state regulation mechanisms [4]. While such movements have long driven the French professional and scientific world [5], the general population remains largely unaware of the stakes TGD individuals face both on a political and health level. In this context, promoting TGD individuals’ health would require developing literacy—a central element of health promotion [6]. However, knowledge is a politically situated construct influenced by power dynamics [7,8]. This is especially relevant in a state of epistemic uncertainty [8] and systemic epistemic injustice [9]. Therefore, knowledge dissemination is placed in a political context reflecting social power dynamics which may affect research and scientific communication [8]. As a result, the TGD population’s health is directly impacted by conditions of knowledge production and interpretation [10] both shaped by research ideologies and, ultimately, paradigms [9]. Achieving significant health results may require moving away from 19th-century paradigms [11] and therefore it would contribute to a paradigm shift [9,11,12].

In the initial French ICD-11 publication, the depsychopathologized diagnosis was mistakenly translated as ‘gender dysphoria’. It has since been corrected to ‘gender incongruence’ (Content Enhancement Proposal #2H2N). Given the previously described context, such errors—implying the pursuit of psychopathologization—could have a disastrous and confusing impact on the political debate. This translation error on one of the emblematic changes of the revision motivates us to analyze the revision of ICD-11 through its French translation. To this end, we will present the conceptual and political stakes of ICD-11 for TGD health, power dynamics in participatory research, and the methodology used for the ICD-11 French translation.

Depsychopathologization of TGD communities followed decades of political activism [13]. It also conceptually followed the depathologization of homosexuality, effective in 1990 for the tenth revision [8]. Politics and ethics led the World Health Organization (WHO) to reconsider the pathologization of TGD persons for ICD-11, where these issues were reconceptualized as a nosographic question opposing two classifications. Because psychopathologization was seen as carrying an unfounded stigma, the WHO working group suggested removing the diagnoses from the chapter on mental and behavioral disorders [14]. This proposal was supported by six field studies highlighting that distress and dysfunction were predicted by social exclusion and violence, not by being transgender [15]. In addition, the global analysis provided evidence that including distress in the diagnostic model would make it less effective [15]. Although pathologization is considered a form of discrimination [16], the WHO has decided to maintain it to protect services access [17]. This has been widely criticized by TGD associations demanding complete depathologization, especially for children [18].

One of the field studies was conducted in Lille (France) by a WHO collaborative centre in mental health, in collaboration with Maison Dispersée de Santé (MDS), a multidisciplinary primary healthcare provider, based on risk-reduction, the free and informed consent models [19], and working closely with local TGD organizations [3,20]. Participation of concerned individuals is one of the center’s terms of reference, and as such, the French study was built around a participatory steering committee, which notably led to the collection of additional socio-cultural data [20]. However, these data were scarcely exploited in the initial paper, nor were they ever developed afterwards. By requiring the collaboration of people with a variety of social status, participatory studies face power struggles during the research process [21]. As a result, experts by experience are at risk of eviction especially prior to the study’s implementation [21]. This was the case in the initial study, under the guise of technicity [22]. Additionally, collected data were not used past the initial publication and access to data was gated behind administrative procedures. Not exploiting insufficiently collected data on a population with high health needs raises the ethical question of research integrity. More importantly, within TGD health research, these issues are the field of power struggles due to systemic transphobia in research pipelines [9,22]. Peer-led research, such as survivor research [23], appears both as a solution to power issues, and as a way of enhancing the conceptual and ecological benefits of participatory research [21,24].

The translation of ICD-11 from English to French was provided by another WHO collaborating centre on international classifications. The WHO translation process is based on back translation [25], meaning a one-to-one equivalence between terms and concepts in different languages does exist [26]. This approach is disproportionately used in medical research and poses difficulties, particularly in considering the socio-cultural context [26]—one of the WHO’s focus areas for ICD-11 [27]. For the first time, the initial translation step was assisted by an initial phase of neural machine translation (NMT) where ICD-11 was automatically translated by natural language processing (NLP) to speed up and improve the translation process [28]. However, this method does not address the issue of systemic bias amplification [29], widely documented for gender [30] and race [31], or the way the translation team organized to address it.

This paper explores the epistemic challenge of pathologizing TGD individuals through the analysis of unexploited data. Then, it focuses on the power issues in participatory research through an approach directly supervised by a TGD researcher. Finally, it draws on this approach to address systemic discrimination within a socio-cultural context. The research objectives were to analyze the socio-cultural data available and the French ICD-11 translation process.

## 2. Materials and Methods

### 2.1. Epistemic Challenges

This paper focused on knowledge derived from the subjective experience of a phenomenon, known as experiential [32]. Knowledge is socially constructed through language using production and interpretation within an ideological framework [10,24]. Since analyzing knowledge requires an understanding of the socio-cultural contexts and involves the subjectivity of the analysts, controlling this subjectivity and putting it at the service of the analysis becomes a methodological challenge. This intervention of the analyst’s subjectivity in research is not specific to the exploration of experiential knowledge and, more broadly, questions the impact of power differences in research dynamics with possible epistemic or discriminatory ramifications [9,21], especially regarding transgender healthcare [8]. To reduce the biases associated with power differences in research, this paper is based on a participatory approach and, more specifically, in line with research directed by the concerned persons, known as survivor research [23]. Design, implementation, analyses, and interpretations were under the direct lead of a TGD researcher (A.B.), with actual experience in the French context, the initial research, and the ICD-11 process [3,8,20,33,34,35]. Analyses were conducted with a cisgender researcher with actual experience in the ICD-11 process (M.G.) [8,33,35]. Ideally, such an analysis should be based on a transparent, participatory, and intersectional approach. Given the time delay between the study and this analysis, as well as the change of research team, it was not possible to include the participants. Nevertheless, this approach aims at improving the quality of analysis by drawing on the subjective experience of analysts and facilitates the use of heuristic approaches while maintaining strong ecological validity [21,24].

### 2.2. Ethical Conformity

The protocol complied with the Helsinki Declaration of 1975, as revised in 2008, and the WHO Good Clinical Research Practice guidelines. The research received approval from the biomedical research French authorities: the *Comité de Protection des Personnes Nord-Ouest IV*, the *Comité Consultatif sur le traitement de l’information en matière de Recherche dans le domaine de la Santé*, and the *Commission Nationale de l’Informatique et des Libertés*. Informed written consent was obtained from every participant.

All data were accessed through a free of charge convention with *EPSM Lille-Métropole*.

### 2.3. Materials

During 2017, seventy-two transgender service users at the MDS, aged 18–50, voluntarily participated in the French study. They were interviewed using first a questionnaire, translated and adapted from the one used in the initial study in Mexico [36], and an additional one specific to France and focused on participants’ insights [20]. Answers to the first questionnaire were used to gather quantitative socio-demographic information on participants, while the second one provided all the qualitative data on the French translation.

The initial paper excluded three “queer” individuals from the analyses [20]. As this article focuses on the entire sample, we included available descriptive data on participants: age, relationship and family status, economic status, education, age of first access to medical transition (hormones or surgery), and gender via a two-step method [37].

Participants’ expertise was gathered, as part of the additional questionnaire, through four consecutive questions: “1. The WHO proposes to designate gender change by the term ‘gender incongruence’. If you had to translate it into French, how would you translate it?”; “2. Does this term seem relevant to you as a way of referring to yourself and people in transition?”; “3. Why?”; “4. Do you have any ideas about how best to refer to gender change?”. The first question was semi-closed and proposed six translations to the participants while leaving the possibility for their own, the three following ones were open-ended. All questions were non-mandatory.

In a final step, we included both English and French finalized versions of ICD-11 descriptions for both diagnoses (HA60—gender incongruence of adolescence or adulthood and HA61—gender incongruence of childhood) and their parent category (gender incongruence).

### 2.4. Analyses

All statistical analyses were performed using R v4.2.0. Descriptive statistics included frequencies (in percentages) for categorical variables, and means, standard deviations (SD), and range for continuous variables. For open-ended data, corpora were created using Notepad++ v8.3.3 and the two authors (A.B. and M.G.) applied a heuristic thematic analysis methodology to code and categorize answers [38]. Disagreements were anticipated to be resolved through discussion and consensus building, but none occurred.

In the initial paper, diagnosis was approached from a functional perspective: as a health tool enabling access to care, and therefore the authors used a top-down approach of morphological analysis to highlight upon which morpheme the tool’s name should be built [20]. The main goal was to support the WHO’s proposal of depsychopathologization and no clear goal was set for the analysis of original French participatory data [20]. Here, the participatory and bottom-up approach allowed us to take distance from these conceptual and methodological frameworks. Our focus was set on letting the analysis angle emerge from data.

Official descriptions were translated from English to French to discuss discrepancies with participants’ data. In accordance with experts’ inputs and practices outside medical research [26], we chose to move away from back translation used by the WHO. The team and translation process were embedded in the research from the beginning. We used a parallel translation from two native speaking translators (A.B. and M.G.) with a final revision step where translation discrepancies were resolved by consensus building within the entire research team. This methodology was proposed to improve the validity of translations for target audiences and socio-cultural contexts, and to reduce biases caused by back translation [26]. This approach reflects a paradigm shift—not considering languages as strictly equivalent but emphasizing the ecological validity and acceptability of translation to focus on health. Both translator–researchers are native speakers of French (continental France), have excellent English skills, and are familiar with concepts specific to health and transgender individuals [25]. The translation also drew on academic knowledge of linguistics (G.M.), experiential knowledge of being a transgender person (A.B.), and first-hand experience of parts of the ICD revision process (A.B. and M.G.).

## 3. Results

### 3.1. Description of Participants

Socio-demographic features of participants are presented in Table 1, original answers and translations are available in Supplementary Table S1. No participant accessed surgery before or without hormonal treatment.

The question on the appropriate French translation of gender incongruence was answered by 43 (59.7%) participants, of which 25 (58.1%) chose to translate differently than the official translation, leaving it approved by 25.0% of total participants. No participant proposed an original translation. The 42 (58.3%) who expressed their opinion on the relevancy of ‘gender incongruence’ to refer to themselves and other transgender individuals gave 11 (26.2%) positive, 11 negative (26.2%), and 20 neutral (47.6%) answers. While the question was open-ended, participants spontaneously used these three categories. In the end, only 3 (4.2%) participants gave positive feedback on the official translation.

### 3.2. Participants’ Propositions

The participants gave 69 justifications for their proposals—a 95.8% response rate—organized around two central components: usage and concepts. Some participants chose to complete their explanations in the open-ended comments at the end of the study. For this reason, we chose to include them in the final analysis, adding 19 rationales. This brought the total number of submissions to 88. Usage was the most discussed aspect, and 72 (81.8%) arguments were classified by researchers into three themes, using eight distinct codes:

- the term was deemed unsuitable by 33 (45.8%) arguments: too complex (*n* = 16; 48.5%), vague (*n* = 10; 30.3%), dated (*n* = 5; 15.2%), or unpronounceable (*n* = 2; 6.1%): “Incongruence is a word that is not related to current events, it is not new enough, not used in the common vocabulary. We need something simpler and especially not word-for-word which is the worst thing to do. It seems to imply out of place or off topic. The cultural aspect is not taken into account here because incongruence is a word that does not pass in French”;
- the negative connotation of the term was highlighted by 22 (30.6%) arguments, 17 (77.3%) considered it pejorative, and others associated it with abnormality: “When I hear incongruence, I hear abnormal. I find it stigmatizing in French. It’s a cis-normative term”;
- the remaining arguments (*n* = 17; 23.6%) expressed disapproval with the term, whether because of personal dislike (*n* = 13; 76.5%) or for its medical consonance: “It’s still a medical term. I don’t like medical terms because identity is not medical. Besides, we don’t say a cis person is gender congruent”.

Out of the 88 arguments, 22 (25.0%) focused on concepts:

- whether the diagnosis should refer to medical transition or an individual’s identity was discussed in 9 (40.9%) arguments: “Good before transition but during, it’s average. It’d be more appropriate for people before their transition because incongruence is tied to the moment when you don’t really know, so before coming out. When you’re sure, you’re no longer incongruent”;
- the diagnostic utility was directly challenged in 6 (27.3%) arguments: “Not naming us is better, I don’t want to be seen as a trans person but as a man”;
- the switch from sex to gender was discussed in 4 (18.2%) arguments: “Because this term is not about ‘sexual’, it is not associated with sexual activities which have nothing to do with us”;
- the term was discussed as related to the body in 3 (13.6%) arguments, with mixed feedback: “It’s a term showing the body does not follow”.

Outside of this usage and concepts framework, 16 (18.2%) arguments were on a higher meta level where 12 (75.0%) expressed overall satisfaction regarding their involvement, ranging from noting an improvement to feeling satisfied, and 4 (25.0%) pointed to the futility of creating new terms in an already saturated field.

Alternative propositions (*n* = 71; 98.6%) from participants are presented in Table 2. Conceptually, they focus on expressing identity (*n* = 31; 43.7%), medical transition (*n* = 22; 31.0%), or pathology (*n* = 13; 18.3%). At the end of the questionnaire, 19 (26.8%) participants spontaneously mentioned terms they found inappropriate and why:

- “transexual” was cited 13 (68.4%) times as a term not to use because it confuses sex and gender (*n* = 7; 53.8%) and is pejorative (4; 30.8%);
- “sex/gender change” because gender doesn’t change (*n* = 2; 66.6%) and sex change is a cis-normative narrative focusing on sexual organs;
- “trans*” and “transgender” with the only justification being that “the sound ‘trans’ reminds of porn” (*n* = 1; 33.3%).

### 3.3. Translation Process of ICD-11 from English to French

The final translation step highlighted several discrepancies, as summarized in Table 3. We found that some English ICD-11 terms lacked a single referential translation in French. Those terms and translation decisions contributed to a loss of the original meaning and possibly to a total loss of meaning on a sentence scale. Consensus building did not support that variation in terminology was related to contextual use adaptations, and highlighted several instances where the French version translated facts into feelings. For example, “experienced gender” is translated as “felt gender” or “gender with which a person identifies”, and “anticipated” as “supposed” in “anticipated […] sex characteristics”. Moreover, “incongruence” was systematically translated by “discordance” besides the diagnosis itself.

## 4. Discussion

Our methodology is well adapted to the analysis of experiential knowledge. However, generalizing results faces limitations as the geographic sample is not representative of the whole French-speaking population, and as participation in scientific studies involves biases in representation. Rather than providing direct answers, this paper gives access to trans perspectives on the WHO data set of ICD-11 translation. Key points on translation and revision are discussed below.

The French correction of “gender incongruence” does not show up favorably in the results, both in terms of usage and representation. The participants report that when used in context, the term clashes with everyday language, making it unintelligible and highlighted. This highlight is negatively connoted by two distinct mechanisms: derogatory use tied to abnormality and dislike tied to pathologization. These notions of abnormality and pathology are brought together under the same theme among transphobic microaggressions, which may explain their negative connotation [16].

These negative connotations question the WHO’s decision to keep pathologizing TGD individuals, particularly when participants question whether the focus of the diagnosis should be on identity or access to medical transition. Some participants suggested giving the term a corporal dimension to put the emphasis on depsychopathologization, while other participants suggested abandoning the concept altogether. Although dropping the concept was also supported by part of the initial research team, this did not lead to a participatory dialogue with the participants [22]. These elements did not appear in the initial publication [20]. If limiting the diagnosis to medical transition would impact, for example, health promotion strategies addressing TGD populations’ vulnerabilities [39], it is unrealistic to expect a medical diagnosis of the entire TGD population—especially given that recent studies show that only one in 15 transgender individuals is actually diagnosed [40]. Maintaining a gatekeeping of health services behind a diagnosis thus contributes to maintaining a community within a community and, in effect, medical power over the lives of TGD individuals [8].

This power struggle is apparent in the interaction of sex and gender in the research—a recurrent theme in participants’ discourse. Medical research does not differentiate between the two, hindering the understanding of the phenomena and its application to trans health, where research is largely lacking [39]. Field studies conducted as part of the ICD-11 revision process on diagnosis associated with the TGD population [15] followed a two-step method of sex and gender data collection, as recommended in literature [37]. In this paper, the analysis was based on the questionnaire results. Table 1 shows groups analyzed in the initial paper [20] were not consistent with participants’ responses. After further investigation, the initial analyses relied on a retrospective gender recategorization of gender-diverse participants into a binary model. In practice, resulting categories are based on sex and not gender, with the additional drawback of discriminatorily excluding three persons from the analysis based on their gender expression. This step does not appear in the paper [20], is not based on up-to-date knowledge of health behaviors of gender diverse individuals [41], contributes to erase gender diversity from research, demonstrates the impact of a cis-normative view of gender expression on research methodology [22], and in this way, supports the hypothesis of a disconnection between the research team and other stakeholders [22].

The French translation of ICD-11 followed a three-step process: a NLP pipeline provides an infrastructure for a second translation step [28], followed by a back translation with harmonization of term equivalence [25,26]. The NLP step, which relies on NMT for short or long texts, aims to facilitate and accelerate the translation process [28]. Contrary to what we anticipated, biases exist in the final product and relate to psychopathologization of TGD individuals. The text evokes a NMT translation without human proofreading and the final descriptions biases would then be entirely caused by the NLP step. This is consistent with the automated pipeline being based on the fairseq toolkit [42], pretrained on reference corpora, combining official and press documents, and then retrained on a corpus of existing medical terminologies [28]. It is therefore not surprising psychopathological microaggressions [16] appear in the translation. Given current knowledge on automatic data debiasing [29,30], and beyond facilitating and accelerating the translation process, applying NLP techniques to medical terminologies places the responsibility of intersectionally debiasing the output on the translation team. Otherwise, health gaps would be automatically reproduced and amplified within medical classifications with general practical consequences on the health of communities [27].

The results point to failures of the translation stage larger than the initial error in the diagnosis itself and question the translation team and the TGD health experts’ socio-cultural competence. Moreover, we question back translation as some terms do not have one-to-one equivalences and these discrepancies cannot be explained by usage in context. We can also note that, outside of diagnosis, the term “incongruence” has been translated as “discordance” in the French version of ICD-11. Usage in context led to “incongruence” being translated into Spanish as “discordance”, and our findings suggest a similar situation in French.

## 5. Conclusions

Ultimately, these results question the relevance and implementation of the depsychopathologization process carried out by the WHO for ICD-11 and highlight failures at all three stages of the official French translation. Power issues have an impact on the production of knowledge on TGD individuals both within research or public debates and on a global or national scale. While mechanisms vary, they all relate to epistemic injustice and notably to maintaining wanted ignorance [9], and their impact is systematically to the detriment of TGD individuals.

Involving TGD populations in all stages of medical knowledge production processes would allow a unified approach able to develop individualized strategies to face each problem to be proposed. If the possibility of collaborative work is not seized for ICD-11, having individuals with personal stakes involved in politicized research areas appears even more necessary today.

## Figures and Tables

**Table 1:** Socio-demographic features.

	Total Sample (*n* = 72)		
	*mean (SD; range)*		
**Age (years)**	27.7 (9.7; 18–50)		
**Monthly income (euros)**	921.0 (1100.4; 0–6000)		
**Education (years)**	13.8 (2.5; 9–20)		
**Age of access to medical transition (years, *n* = 60)**	25.2 (9.0; 14–50)		
	*n (%)*		
**Marital status—single**	52 (72.2%)		
**Children—none**	65 (90.3%)		
**Sex assigned at birth—female**	28 (38.9%)		
**Sex assigned at birth—male**	44 (61.1%)		
**Sex assigned at birth—intersex**	0 (0.0%)		
		Sex assigned at birth—female	Sex assigned at birth—male
**Gender—woman**	34 (47.2%)	0 (0.0%)	34 (77.3%)
**Gender—man**	22 (30.6%)	22 (78.6%)	0 (0.0%)
**Gender—transwoman/transman**	8 (11.1%)	4 (14.3%)	4 (9.1%)
**Gender—other**	8 (11.1%)	2 (7.1%)	6 (13.6%)

**Table 2:** Alternative propositions of terms for ICD-11.

Propositions	Literal Translations	Occurrences (*n* = 71)
Transgenre ^1^	Transgender	15 (21.1%)
Transidentité ^1^	Transidentity	11 (15.5%)
Transition ^2^	Transition	9 (12.7%)
Dysphorie de genre ^3^	Gender dysphoria	8 (11.3%)
Changement de genre ^2^	Gender change	3 (4.2%)
Parcours transgenre ^2^	Transgender path	3 (4.2%)
Dysphorie ^3^	Dysphoria	2 (2.8%)
Acceptation de l’identité ^2^	Acceptance of identity	1 (1.4%)
Adaptation du genre ^2^	Gender adaptation	1 (1.4%)
Affirmation de l’identité de genre ^2^	Affirmation of gender identity	1 (1.4%)
Affirmation de l’identité personnelle ^2^	Affirmation of personal identity	1 (1.4%)
Binaire	Binary	1 (1.4%)
Chimère	Chimera	1 (1.4%)
Diversité du/des genre(s) ^1^	Gender diversity	1 (1.4%)
Homme-Fleur	Man-flower	1 (1.4%)
Inadaptation du genre ^3^	Gender inadequacy	1 (1.4%)
Incongruence corporelle du genre	Gender bodily incongruence	1 (1.4%)
Personne trans’ ^1^	Trans person	1 (1.4%)
Questionnement sur la transidentité ^1^	Questioning transidentity	1 (1.4%)
Réassignation ^2^	Reassignment	1 (1.4%)
Réassigné de l’identité ^1^	Identity-reassigned	1 (1.4%)
*Shemale*	Shemale	1 (1.4%)
Singularité de genre ^1^	Gender Singularity	1 (1.4%)
Transsexualisme ^3^	Transsexualism	1 (1.4%)
Transsexuel ^3^	Transsexual	1 (1.4%)
Transformation de l’identité ^2^	Identity transformation	1 (1.4%)
Transition de genre social ^2^	Social gender transition	1 (1.4%)

These propositions were categorized as expressing: ^1^ identity, ^2^ medical transition, or ^3^ pathology.

**Table 3:** Discrepancies between translations.

English Terms	French Terms (*n*)	Proposed Terms (*n*)
incongruence	discordance (3); sentiment de discordance (1)	incongruence (4) *
experienced gender	genre auquel une personne s’identifie (2); genre ressenti (2); sexe vécu (1); genre vécu (1)	genre vécu (6)
gender variant	qui varient en fonction du sexe (3)	de variation de genre (3)
gender	sexe (1)	genre (1)
anticipated […] sex characteristics	caractéristiques sexuelles […] supposées (2)	caractéristiques sexuelles […] anticipées (2)

* The term incongruence has been translated literally, but this choice would have to be questioned in the event of modification of the diagnostic terms in French.

## Data Availability

Data were accessed by a convention with the World Health Organization Centre for research and training in mental health (*EPSM Lille-Métropole*) which does not allow public release of materials. Initial study data can be accessed upon request to the owner.

## Supplementary Materials

The following supporting information can be downloaded at: https://www.mdpi.com/article/10.3390/ijerph191911983/s1, Table S1: Original French answers and English translations.
