# Supplementary material for: Trans Expertise and the Revision and Translation of ICD-11: An Analysis of Available French Data from a Trans Perspective
Source: Int J Environ Res Public Health. 2022 Sep 22;19(19):11983. doi: 10.3390/ijerph191911983 (PMC9564459; doi:10.3390/ijerph191911983)
Supplement: Supplementary file 1 [file ijerph-19-11983-s001.zip › ijerph-1884021-supplementary.pdf]

Table S1. Original French answers and English translations.

French answers	English translations
Incongruence est un mot qui n'est pas en rapport avec l'actualité, c'est un mot pas assez neuf. Pas utilisé dans le vocabulaire commun. Il faut quelques chose de plus simple et surtout pas du mot à mot, c'est la pire des choses à faire. Semble vouloir dire déplacé ou pas dans le sujet/déplacé. L'aspect culturel n'est pas pris en compte car incongruence est un mot qui ne passe pas en français.	"Incongruence is a word that is not related to current events, it is not new enough, not used in the common vocabulary. We need something simpler and especially not word-for-word which is the worst thing to do. It seems to imply out of place or off topic. The cultural aspect is not taken into account here because incongruence is a word that does not pass in French"
Quand j'entends incongruence, j'entends anormal. Je trouve ça stigmatisant en français.C'est un terme cis-normatif.	"When I hear incongruence, I hear abnormal. I find it stigmatizing in French. It's a cis-normative term"
Ca reste un terme médical. Je n'aime pas les termes médicaux car l'identité n'est pas médical. D'ailleurs on ne dit pas d'une personne sis qu'elle a une congruence de genre.	"It's still a medical term. I don't like medical terms because identity is not medical. Besides, we don't say a cis person is gender congruent"
Bien pour avant la transition mais pendant la transition c'est moyen. C'est plus approprié pour les personnes avant leur transition car incongruence est en relation avec la période où on ne sait pas trop donc avant le coming out. Quand on est sûr, on est plus incongruent.	"Good before transition but during, it's average. It'd be more appropriate for people before their transition because incongruence is tied to the moment when you don’t really know, so before coming out. When you’re sure you’re no longer incongruent"
Ne pas nous nommer c'est mieux car je ne veux pas être perçut comme une personne trans mais comme un homme.	"Not naming us is better, I don't want to be seen as a trans person but as a man"
Car ce terme ne parle pas de "sexuel", il n'est pas connoté aux activité aux activités sexuel ce qui n'a rien à voir avec nous	"Because this term is not about ‘sexual’, it is not associated with sexual activities which have nothing to do with us"
C'est un terme qui montre que c'est le corps qui ne suit pas.	“It’s a term showing the body does not follow”
Le son trans rappelle le porno.	“The sound trans reminds of porn”
